# Supplementary material for: Antioxidant Activity of Phenolic Extraction from Different Sweetpotato (Ipomoea batatas (L.) Lam.) Blades and Comparative Transcriptome Analysis Reveals Differentially Expressed Genes of Phenolic Metabolism in Two Genotypes
Source: Genes (Basel). 2022 Jun 16;13(6):1078. doi: 10.3390/genes13061078 (PMC9222414; doi:10.3390/genes13061078)
Supplement: Supplementary file 1 [file genes-13-01078-s001.zip › Table S1.pdf]

Table S1. List of sweetpotato genotypes used

Genotype	Genotype	Genotype
160410	18-6-24	Chaoshu1
170901	CT1-13	18-6-1
18-2-18	18-1-1	18-2-21
18-2-4	Shangshu19	Wan1314-6
18-7-12	161010	18-6-43
Zhongshu1	Yushu1	18-11-5
18-6-47	Ningzi4	XN1408-4
161603	161614	
