# Supplementary material for: Antioxidant Activity of Phenolic Extraction from Different Sweetpotato (Ipomoea batatas (L.) Lam.) Blades and Comparative Transcriptome Analysis Reveals Differentially Expressed Genes of Phenolic Metabolism in Two Genotypes
Source: Genes (Basel). 2022 Jun 16;13(6):1078. doi: 10.3390/genes13061078 (PMC9222414; doi:10.3390/genes13061078)
Supplement: Supplementary file 1 [file genes-13-01078-s001.zip › Table S2.pdf]

Table S2. Primers used in qRT-PCR

Gene ID	forward primers for q-RTPCR (5'-3')	reverse primers for q-RTPCR (5'-3')
g10319	TGTACTCGGTGAGGGAGG	AAACTAACATCCCTGGACTC
g24989	GCTTTGTCCAATCCATGATC	CTCACGGAGCAACAAAATAG
g24990	ACCATTGCCATTGTCAAC	CTGGGAGACAACAAAAGACA
g26782	CCCACTAGCCTTTTTCCA	GCCTATGGTCCAACCAAG
g29801	TGAACACTGCCCTGGACC	GCTTAGCTTAGCTTCCTGG
g29802	ACTGCCATAGACCGCCTTA	ACGCTAAACCTCTCACACT
g34957	TGCTTGTTCCCTAGCCTT	CCGCAGTACAAAACCTTG
g43253	TTAACAAAGCCTTGGCCG	ACAGTGCAACTCCAAGAAG
g553	TCCCCAACTTCTCTCTCTCT	GAGCTGTGAGTAGCAAAGAG
g57987	CTCTCCTCCCTGTTCTTCG	CACCATGCGCTTCACTTC
IbACTIN	CTGGTGTTATGGTTGGGATGG	GGGGTGCCTCGGTAAGAAG
