# Supplementary material for: Antioxidant Activity of Phenolic Extraction from Different Sweetpotato (Ipomoea batatas (L.) Lam.) Blades and Comparative Transcriptome Analysis Reveals Differentially Expressed Genes of Phenolic Metabolism in Two Genotypes
Source: Genes (Basel). 2022 Jun 16;13(6):1078. doi: 10.3390/genes13061078 (PMC9222414; doi:10.3390/genes13061078)
Supplement: Supplementary file 1 [file genes-13-01078-s001.zip › table S3.pdf]

Table S3. Antioxidant activity,TPC and TFC of blade in different sweetpotato genotypes

Genotypes	TFC （mg RE/g）	TPC （mg GAE/g）	ABTS+ （mg TE/g）	DPPH· （mg TE/g）	FRAP （mg TE/g）
160410	4.91±0.81cdefg	11.80±2.00defg	22.52±2.34abc	9.54±2.60h	43.24±0.52cd
170901	4.94±1.00cdefg	9.01±2.63fgh	15.19±4.72cde	10.17±8.61gh	25.37±0.78ghij
18-2-18	4.91±1.49cdefg	7.86±1.80ghi	22.02±2.08abc	4.57±5.58ij	29.25±0.57fghi
18-2-4	6.22±1.28bc	15.44±3.65cde	26.82±8.27a	13.40±10.68ef	52.11±0.85bc
18-7-12	4.94±1.14cdefg	15.62±3.61cde	21.84±6.81abc	15.33±6.70cde	39.85±1.59de
Zhongshu1	3.58±0.10fg	4.45±1.77hi	4.59±1.66f	5.73±2.41ij	10.60±1.39m
18-6-47	5.41±0.51cdef	11.92±2.97defg	11.94±4.06def	11.82±4.83fg	20.27±0.28ijkl
161603	3.66±0.13fg	4.26±1.23hi	7.26±2.53f	6.05±3.21ij	13.74±0.97klm
18-6-24	4.05±0.76efg	2.17±0.98i	4.87±1.83f	5.46±2.57ij	9.77±1.41m
CT1-13	4.22±0.64defg	6.72±2.13ghi	9.44±2.90ef	6.33±3.87i	20.15±1.08ijkl
18-1-1	5.96±0.56bcd	17.3±6.33cde	19.58±3.63abc	14.02±3.22def	50.34±1.48bc
Shangshu19	4.44±1.05cdefg	6.22±0.911ghi	5.23±1.054f	3.93±2.14j	9.64±0.56m
161010	4.73±0.56cdefg	11.28±2.59efg	17.64±3.46bcd	11.75±3.66fg	33.29±1.03efg
Yushu1	7.16±0.49b	17.35±2.11bcd	24.84±3.33ab	13.32±7.09ef	55.62±2.05b
Ningzi4	4.30±0.77defg	13.75±3.92cdef	17.80±3.59bcd	12.59±5.98f	23.71±0.51ghij
161614	5.58±0.14cde	15.29±1.79cde	27.13±6.00a	13.50±7.31ef	36.89±1.71def
Chaoshu1	3.60±0.93fg	6.09±2.20ghi	6.99±2.36f	8.69±2.52h	10.01±1.24m
18-6-1	4.75±0.30cdefg	7.98±2.19ghi	11.98±2.54def	9.20±2.64h	17.48±1.54jklm
18-2-21	5.32±2.0cdef	22.50±3.92b	14.90±4.15cde	20.76±4.46a	24.93±0.89ghij
Wan1314-6	10.73±2.0a	28.32±3.63a	25.10±2.48ab	15.93±5.40cd	85.51±2.13a
18-6-43	3.07±0.07g	5.23±1.3lhi	6.00±1.97f	4.66±2.71ij	12.51±1.07lm
18-11-5	5.42±0.51bcde	11.91±2.97bc	12.07±5.37def	18.55±3.42b	22.35±1.17hijk
XN1408-4	6.04±0.12bcd	16.99±2.88bcde	22.10±5.61abc	16.36±6.01c	31.10±1.32efgh

Note: The same letters in same column indicated an insignificant mean difference; The different letters in same columndifferent lower-case letters indicated that the mean difference reached a significant level of $p \leq 0.05$.
