# Supplementary material for: Antioxidant Activity of Phenolic Extraction from Different Sweetpotato (Ipomoea batatas (L.) Lam.) Blades and Comparative Transcriptome Analysis Reveals Differentially Expressed Genes of Phenolic Metabolism in Two Genotypes
Source: Genes (Basel). 2022 Jun 16;13(6):1078. doi: 10.3390/genes13061078 (PMC9222414; doi:10.3390/genes13061078)
Supplement: Supplementary file 1 [file genes-13-01078-s001.zip › Table S4.pdf]

Table S4. Up-regulated genes and annotation of the phenylpropanoid biosynthetic pathway

Gene ID	Genes	Enzyme ID
g57987	*PAL*	EC:4.3.1.24
g26782	*HCT*	EC:2.3.1.133
g16725、g62049	*F5′H*	EC:1.14.14
g31303、g31304	*F3′H*	EC:1.14.14.82
g1027	*CHS*	EC:2.3.1.74
g3586	*CHI*	EC:5.5.16
g553、g43253	*CCoAOMT1*	EC:2.1.1.104
g712、g29801、g29802、g34957	*CAD*	EC:1.1.1.195
g10319	*4CL*	EC:6.2.1.12
g26014	*ALDH2C4*	EC:1.2.1.68
g1369	*UGT72E1*	EC:2.4.1.111
g1383	*UGT72E2*	EC:2.4.1.111
g18287、g1126、g24562、g17001、g48738、g24563、g17013、g24561、g8745	*PRX52*	EC:1.11.1.7
g24989、g24990	*POD*	EC:1.11.1.7
g3595	*F12L6.8*	EC:3.4.24.59
